# Metabolic Reprogramming of Nasal Airway Epithelial Cells Following Infant Respiratory Syncytial Virus Infection

**DOI:** 10.3390/v13102055

**Published:** 2021-10-13

**Authors:** Andrew R. Connelly, Brian M. Jeong, Mackenzie E. Coden, Jacob Y. Cao, Tatiana Chirkova, Christian Rosas-Salazar, Jacqueline-Yvonne Cephus, Larry J. Anderson, Dawn C. Newcomb, Tina V. Hartert, Sergejs Berdnikovs

**Affiliations:** 1 Department of Medicine, Northwestern University Feinberg School of Medicine, Chicago, IL 60611, USA; andrew.connelly@northwestern.edu (A.R.C.); brian.jeong@northwestern.edu (B.M.J.); mackenzie.coden@northwestern.edu (M.E.C.); jycao12@gmail.com (J.Y.C.); 2 Department of Pediatrics, Emory University School of Medicine and Children’s Healthcare of Atlanta, Atlanta, GA 30322, USA; tania.chirkova@emory.edu (T.C.); larry.anderson@emory.edu (L.J.A.); 3 Department of Medicine, Vanderbilt University Medical Center, Nashville, TN 37232, USA; c.rosas.salazar@vumc.org (C.R.-S.); jacqueline.y.cephus@Vanderbilt.Edu (J.-Y.C.); dawn.newcomb@vumc.org (D.C.N.); 4 Department of Pathology, Microbiology and Immunology, Vanderbilt University Medical Center, Nashville, TN 37203, USA; 5 Department of Pediatrics, Vanderbilt University Medical Center, Nashville, TN 37203, USA

**Keywords:** respiratory syncytial virus, airway epithelial cells, metabolism, glucose, infant

## Abstract

Respiratory syncytial virus (RSV) is a seasonal mucosal pathogen that infects the ciliated respiratory epithelium and results in the most severe morbidity in the first six months of life. RSV is a common cause of acute respiratory infection during infancy and is an important early-life risk factor strongly associated with asthma development. While this association has been repeatedly demonstrated, limited progress has been made on the mechanistic understanding in humans of the contribution of infant RSV infection to airway epithelial dysfunction. An active infection of epithelial cells with RSV in vitro results in heightened central metabolism and overall hypermetabolic state; however, little is known about whether natural infection with RSV in vivo results in lasting metabolic reprogramming of the airway epithelium in infancy. To address this gap, we performed functional metabolomics, 13C glucose metabolic flux analysis, and RNA-seq gene expression analysis of nasal airway epithelial cells (NAECs) sampled from infants between 2–3 years of age, with RSV infection or not during the first year of life. We found that RSV infection in infancy was associated with lasting epithelial metabolic reprogramming, which was characterized by (1) significant increase in glucose uptake and differential utilization of glucose by epithelium; (2) altered preferences for metabolism of several carbon and energy sources; and (3) significant sexual dimorphism in metabolic parameters, with RSV-induced metabolic changes most pronounced in male epithelium. In summary, our study supports the proposed phenomenon of metabolic reprogramming of epithelial cells associated with RSV infection in infancy and opens exciting new venues for pursuing mechanisms of RSV-induced epithelial barrier dysfunction in early life.

## 1. Introduction

Respiratory syncytial virus (RSV) is a leading global cause of respiratory disease in infants [1]. It results in its greatest morbidity in the first six months of life and is a common cause of acute respiratory infection during infancy. RSV is a seasonal mucosal pathogen that infects the ciliated respiratory epithelium of the upper and lower airway as a descending infection, causing disease of variable severity [1,2]. Inappropriate or dysregulated responses to RSV can be pathogenic, causing disease-enhancing inflammation that contributes to short- and long-term effects. RSV lower respiratory tract infection (LRI) is strongly and consistently associated with wheeze and asthma. Asthma pathogenesis is characterized by a dysfunctional airway epithelial barrier and is caused by complex interactions between genetic and environmental factors, with early-life exposures likely playing a vital role [3,4]. RSV has been shown to be an important and common early-life modifiable asthma risk factor strongly associated with asthma development [5,6,7,8,9,10,11]. While this association has been repeatedly demonstrated for decades, rather limited progress has been made on the mechanistic understanding in humans of the contribution of infant RSV infection on airway epithelial dysfunction.

The modification of host airway epithelial cellular metabolism by early-life exposures is one such overlooked factor despite the central importance of metabolism in regulating development and function of the epithelial barrier [12,13]. Several studies have documented that human RSV infection in vivo and bronchiolitis are accompanied by systemic changes in metabolism [14,15,16,17,18]. Metabolic changes resulting from RSV infection of epithelial cells in vitro have also been previously documented [19,20,21]. Active infection with in vitro RSV increases cellular energetic demands and results in increased activity of mitochondrial and cellular metabolism pathways. These changes largely favor the activity of glycolytic and pentose phosphate pathways [21]. However, despite growing interest in targeting metabolic reprogramming of RSV and influenza infection for therapeutic intervention [20,22], virtually nothing is known about in-vivo metabolic reprogramming of epithelial cells from children with history of RSV infection in infancy (less than one year of age). To address this significant gap in the field, we explored whether infant RSV infection is associated with lasting changes in epithelial metabolism in vivo, which has downstream disruptive potential to contribute to chronic respiratory disease by altering airway epithelial cell development. In this manuscript, using both functional metabolomics and genomics analysis of nasal airway epithelial cells (NAECs) sampled from infants between 2–3 years of age, we demonstrated that RSV infection during the first year of life is associated with epithelial reprogramming characterized by (1) significant changes in epithelial uptake and utilization of glucose; (2) altered preferences for metabolism of several carbon and energy sources; and (3) significant sexual dimorphism in metabolic parameters, with RSV-induced metabolic changes most pronounced in male epithelium.

## 2. Materials and Methods

### 2.1. Study Population

The Infant Susceptibility to Pulmonary Infections and Asthma Following RSV Exposure study (INSPIRE) is a population-based birth cohort of healthy, term children specifically designed to determine the presence vs. absence of RSV infection in the first year of life (infancy). The detailed methods for INSPIRE have been previously reported [23]. The Institutional Review Board of Vanderbilt University approved this study, and one parent of each child provided informed consent for their participation. To understand the impact of RSV infection in infancy (0–12 months of age) on metabolic reprogramming of the airway epithelium, we sampled epithelial cells from children at ages 2–3 years during a well-child visit requiring the absence of signs and symptoms of respiratory illness. Visits were rescheduled if children had any sign or symptom of a respiratory illness. We conducted a nested-cohort study in a subset of children (*n* = 24) enrolled in INSPIRE (6 males with and 6 males without a history of RSV infection during the child’s first RSV season; 6 females with and 6 females without history of RSV infection during the child’s first RSV season). None of the children included in this subset had documented wheeze or an active respiratory infection at the time of sample collection (2–3 years of age). “RSV” group designation in presentation of results refers strictly to natural RSV infection during infancy (1–12 months of age). This allowed us to focus our question of the reprogramming effects of RSV in infancy. Table 1 summarizes demographic and clinical data for this study group in comparison to data from the entire INSPIRE cohort.

### 2.2. Identification of RSV Infected and RSV Uninfected Infants

The INSPIRE cohort included an a-priori-designed nested cohort study of children with and without RSV infection during infancy in whom both peripheral blood mononuclear cells and NAECs were collected in follow-up at 2–3 years of age, specifically designed to compare among children who were and were not infected with RSV during infancy. For the ascertainment of RSV infection in infancy, we first conducted passive and active biweekly surveillance during each child’s first RSV season. In children who met pre-specified criteria for an acute respiratory illness, an in-person visit was conducted, and a nasal wash was collected and tested for RSV using reverse transcription-quantitative polymerase chain reaction (PCR) [23,24]. In all participating children, RSV serum antibody titers were also measured in blood samples at age one year. RSV antibodies were detected by RSV A and B enzyme-linked immunoassay (EIA) for detecting prior infant RSV infection using previously published protocols [25]. Children were then grouped into those infected with RSV vs. not infected with RSV in infancy using a hierarchical categorization with mutually exclusive group membership based on the reverse transcription-quantitative PCR tests and one-year RSV serology.

### 2.3. Human NAECs

NAECs were collected from children first at age 2–3 years during a well-child visit. Screening for signs and symptoms of respiratory illness was done, and visits and collections rescheduled for any sign or symptoms of respiratory illness. For all subjects in this study, NAECs were collected and expanded in culture in a consistent manner. NAECs from all donors were tested in submerged conditions in the same confluency (70–80%), passage, time in culture, and media conditions. NAECs were collected from study subjects by brushing nasal passages using a soft flocked cotton swab (Copan, Murrieta, CA, USA) and placed in DMEM media (HyClone) supplemented with 10% FBS (Sigma, St. Louis, MO, USA). Cells were isolated by gently scraping the swab in the media with sterile forceps, centrifuging at 150× *g* for 5 min and pre-expanding in the conditioned F media on the layer of feeder cells as previously described [26,27]. Briefly, cells were plated on a layer of mitomycin C (Sigma) -treated 3T3 mouse fibroblast feeder cells and grown for 4–5 days in the 1:3 mixture of Ham’s F12/DMEM media (HyClone) supplemented with 5% FBS, 24 µg/mL adenine (Sigma), 0.4 µg/mL hydrocortisone (Sigma), 5 µg/mL Insulin (Sigma), 10 ng/mL EGF (Sigma), 8.4 ng/mL Cholera toxin, and 10 µM ROCK1 inhibitor Y-2763 (Selleck Chemical LLC). Media was changed daily to remove debris and non-viable cells allowing live basal cells to expand. Passage 2 NAEC were collected and cryopreserved in FBS with 10% DMSO. For this study, NAECs from all donors were tested in submerged conditions in the same confluency (70–80%), passage, time in culture, and media conditions. Cell density was confirmed by counting adherent cells in the same field of view for all donors. Minor variation in cell density at this confluency has negligible effects on metabolism since cells do not undergo changes due to contact inhibition. Submerged conditions were used to make cells accessible to functional metabolism studies using nutrient-neutral Biolog M1 media.

### 2.4. Metabolism Assays

To investigate the possible connection between RSV infection and epithelial metabolism, we profiled metabolism of carbohydrate and TCA energy sources in live NAECs cultured in submerged conditions. To measure metabolism of carbon and energy sources, 5000 epithelial cells/well were seeded onto Phenotype Microarray PM-M1 plates (Biolog, Cat #:12111) in Biolog IF-M1 (Biolog, Cat #: 72301) media supplemented with 0.5% dialyzed FCS and 0.3 mM *L*-glutamine. Biolog MB (Biolog, Cat #: 74352) redox dye was added, and live cells were then incubated in an OmniLog automated incubator-reader (Biolog Inc., Hayward, CA, USA) for 24 h at 37 °C. Dye reduction, indicative of energy-rich NADH production, was measured at 590 nm absorbance in 15-minute intervals using OmniLog reader (Biolog Inc., Hayward, CA, USA). Kinetic background values were subtracted, and data were normalized to cells from infants who were not infected with RSV (RSV uninfected). Resulting substrate utilization for NADH energy production was quantified by measuring total area under the curve. All healthy donors were compared to RSV donors by sex. To gain further mechanistic insights into differential utilization of glucose by infant RSV vs. RSV uninfected epithelial cells, we performed 13C glucose fluxomics analysis. In these experiments, 12C glucose (a naturally occurring glucose isotopomer) was replaced by a 13C variant of glucose (not occurring naturally) in culture media for three hours. Following the treatment, proportional incorporation of 13C carbons in downstream metabolites was measured by mass spectrometry analysis.

### 2.5. RNA-Seq

NAECs were lysed, and RNA was immediately extracted using the RNeasy Plus Mini Extraction Kit (Qiagen, Hilden, Germany). RNA quality was assessed using Qubit and BioAnalyzer, and qPCR was conducted using the KAPA library quantification kit and QuantStudio for Illumina (Roche). The sequencing of cDNA libraries was done on an Illumina NovaSeq 6000 (Illumina, San Diego, CA, USA) at a target read depth of approximately 50 million aligned reads per sample.

### 2.6. Bioinformatics and Statistical Analysis

RNA-seq sequenced reads were demultiplexed using bcl2fastq (v 2.17.1.14). Quality control was performed using FastQC. Low-quality reads were discarded using trimmomatic (v 0.33). Reads were aligned using TopHat2 aligner. Read counts were generated using htseq-count. Differential expression analysis was done using the edgeR in R/Bioconductor package. *p*-Values were adjusted for multiple comparisons using Benjamini-Hochberg FDR correction. All computational analysis was performed on genomic nodes of Quest, Northwestern’s High Performance Computing Cluster. Gene ontology (GO biological process) analysis was performed in PANTHER 13.1, using two unranked lists of genes (target and background lists) to generate FDR < 0.05 enrichment. To explore overall metabolic variance patterns in results of Biolog data by sex and RSV status, Principal Component Analysis (PCA) was executed in PAST 2.17 software using covariance matrices and singular value decomposition. For metabolic assays, statistical significance was determined by unpaired *t*-tests. All data are represented as mean ± S.D. Statistical analysis was performed using GraphPad Prism and Systat 13.0 software. An alpha level of 0.05 was used as a significance cut-off.

## 3. Results

### 3.1. NAECs from Infants with History of RSV Infection Show Evidence of Reprogrammed Metabolism

**Figure 1:**
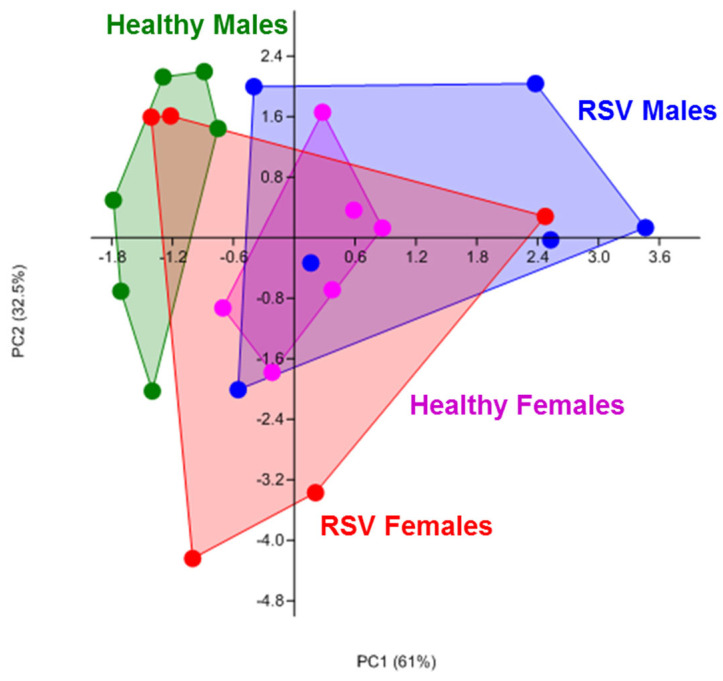
Principal component analysis showing overall differences in carbohydrate metabolism between NAECs collected at age 2–3 years and compared between those with and without RSV infection during infancy and sex, measured as kinetic generation of NADH in a redox colorimetric Biolog assay. Aside from the effect of RSV, note significant separation of male and female healthy control clusters along principal component 1 (horizontal axis). All donors were equalized in culture density, passage, and treatment timelines.

**Figure 2:**
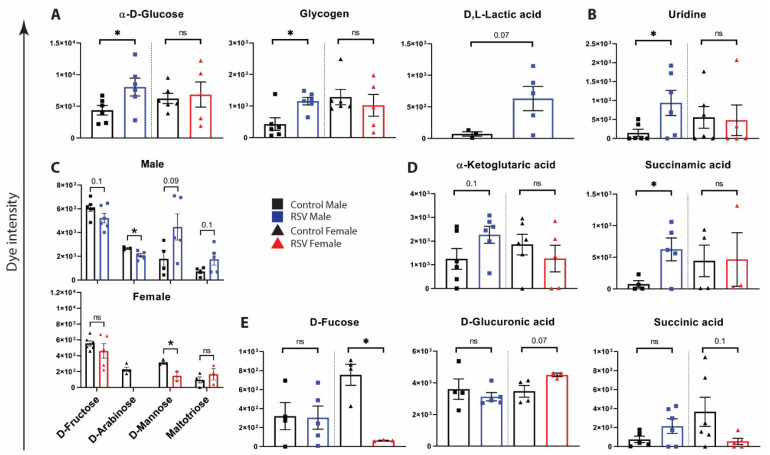
Metabolism of carbon and energy sources by NAECs collected at age 2–3 years and compared between those with or without RSV infection during infancy. Dye intensity refers to colorimetric dye reduction by energy-rich cellular NADH production in Biolog PM-M1 assays (**A**–**D**). RSV-associated metabolic changes are greater in males than females: (**A**) glycolysis; (**B**) uridine metabolism; (**C**) carbohydrate metabolism; (**D**) TCA intermediate metabolism. (**E**) RSV-associated metabolic changes are greater in females than in males. Black = NAEC from male (*n* = 6) or female (*n* = 6) children who were RSV uninfected during infancy (Control). Blue, NAEC from male children who were RSV infected during infancy (RSV M) (*n* = 6). Red, NAEC from female children who were RSV infected during infancy (RSV F) *n* = 6). *, *p* < 0.05 by Student’s *t*-test. Trending but not significant *p*-values are indicated on graphs. n.s., not significant.

To determine if NAECs from infants with a history of RSV infection had different metabolic properties compared to NAEcs from control infants, we conducted a Biolog metabolism assay of carbon and energy sources (PM-M1 phenotype commercial assay (Biolog, Inc.) measuring NADH output from live cells presented with 91 different carbohydrate and TCA cycle intermediate energy sources). Using PCA analysis, the results from this assay suggested that NAECs from children with a history of RSV infection during infancy may be metabolically reprogrammed when sampled at 2–3 years of age (Figure 1). Notably, there were differences in overall patterns of metabolism in NAECs between males and females (Figure 1). Out of 91 metabolites included in the assay, male NAECs had significant changes in metabolism of five energy sources (five additional metabolites showed trends, but were not significant) (Figure 2). Female NAECs had significant changes in metabolism of two energy sources (two additional metabolites showed trends, but were not significant) (Figure 2). Specifically, male NAECs had significantly increased metabolism of glucose, glycogen, uridine, and succinamic acid (derivative of succinate). Male NAECs also had a trend in increase in metabolism of lactic acid (female lactic acid could not be detected), α-ketoglutaric acid, mannose, and maltotriose as well as decreased metabolism of fructose and arabinose. Female NAECs had decreased metabolism of mannose, fucose, and succinic acid and trend in increased consumption of glucuronic acid. In summary, by direct testing of NADH production from various cellular energy sources, the results implicate significant changes in pathways of central importance to cellular metabolism, glycolysis in particular. In addition, male NAECs demonstrated a greater degree of change in energy metabolism than females.

### 3.2. Gene Expression Analysis of NAECs Supports RSV-Associated Reprogramming of Key Metabolic Pathways

**Figure 3:**
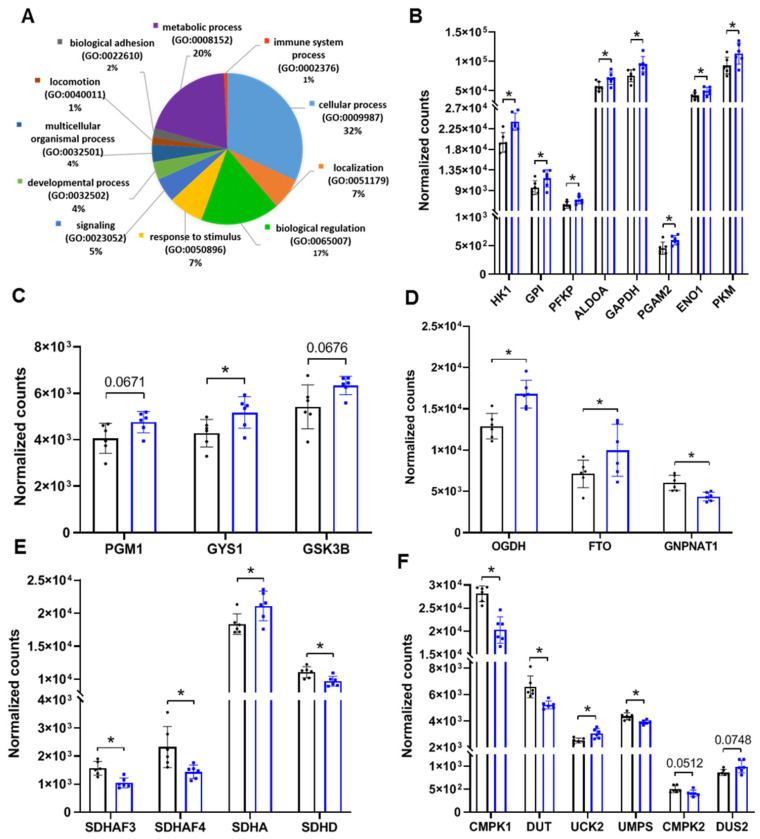
RNA-seq analysis of metabolic markers and pathways in NAECs derived from male children with or without infant RSV infection. (**A**) Biological process analysis of male DEGs from RSV infected vs. RSV uninfected; 20% of DEGs represent changes in biological processes associated with cellular and mitochondrial metabolism (purple portion of the chart). (**B**) Expression of markers of glycolysis. (**C**) Markers of glycosylation and glycogenesis. (**D**) Alpha-ketoglutarate/acetyl-CoA metabolism. (**E**) Succinate metabolism. (**F**) Uridine metabolism. Black bars, healthy controls (*n* = 6); blue bars, male samples with RSV infection in infancy (0–12 months) (*n* = 6). * *p* < 0.05 by FDR-corrected test. Trending but not significant *p*-values are indicated on graphs.

We also performed RNA-seq analysis of matched NAEC samples for which we had metabolic data to determine whether there is genomic level evidence for RSV-associated epithelial metabolic reprogramming. Male NAECs had 966 differentially expressed genes (DEGs) by false-discovery rate (FDR)-adjusted statistical significance (3840 non-adjusted), while female NAECs had 2 DEGs (589 non-adjusted) due to increased intragroup variability. Biological process analysis revealed that 20% of all DEGs in male NAECs reflected significant changes in metabolic processes (Figure 3A). We further interrogated specific male genes reflecting enzymatic activity in pathways associated with metabolic changes highlighted in Figure 2. We found that all genes representing enzymes enabling the glycolytic pathway were significantly upregulated, including hexokinase (*HK1*), glucose-6-phosphate isomerase (*GPI*), phosphofructokinase (*PFKP*), aldolase (*ALDOA*), glyceraldehyde 3-phosphate dehydrogenase (*GAPDH*), phosphoglycerate mutase (*PGAM2*), enolase (*ENO1*), and pyruvate kinase (*PKM*) (Figure 3B). We also found significant increases in the expression of genes associated with glycosylation and glycogenesis (*PGM1*, *GYS1*, *GSK3B*) (Figure 3C), alpha-ketoglutarate/acetyl-CoA metabolism (*OGDH*, *FTO*, *GNPNAT1*), succinate metabolism (*SDHAF3*, *SDHAF4*, *SDHA*, *SDHD*) (Figure 3D), and uridine metabolism (*CMPK1*, *DUT*, *UCK2*, *UMPS*, *CMPK2*, *DUS2*) (Figure 3E). Collectively, genomic analysis supported significant changes in glycolysis and other key metabolic pathways in NAECs from 2–3 year old children who had RSV infection during infancy compared with NAECs from children who were not infected with RSV during infancy.

### 3.3. Sex Differences in NAEC Metabolic Pathways

**Figure 4:**
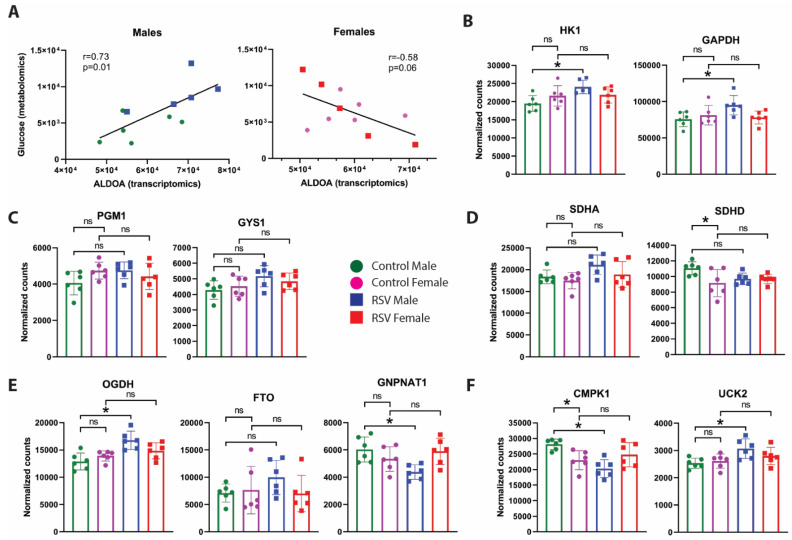
(**A**) Correlations between metabolomic data (α-D-glucose metabolism by Biolog) and transcriptomic data (aldolase expression by RNA-seq) for glucose metabolism specifically. Blue, males; red, females. (**B**–**F**) Direct comparisons of male and female data for key metabolic markers in RNA-seq analysis of metabolic markers and pathways in nasal epithelial cells derived from male with or without history of infant RSV infection. (**B**) Expression of markers of glycolysis. (**C**) Markers of glycosylation and glycogenesis. (**D**) Succinate metabolism. (**E**) Alpha-ketoglutarate/acetyl-CoA metabolism. (**F**) Uridine metabolism. Green = NAEC from male children who were RSV uninfected during infancy (*n* = 6). Purple = NAEC from female children who were RSV uninfected during infancy (*n* = 6). Blue = NAEC from male children who were RSV infected during infancy (*n* = 6). Red = NAEC from female children who were RSV infected during infancy (*n* = 6). * *p* < 0.05 by ANOVA/Tukey multiple testing. ns, not significant.

We further examined whether male and female NAECs have differences in metabolism by gene expression. Glucose NADH conversion (from Biolog assays) and aldolase gene expression (marker of energy-producing glycolysis) were significantly correlated in male NAECs but not female NAECs (Figure 4A), which is consistent between metabolomics and genomic analyses but also demonstrates sex differences. Although there were baseline sex differences in expression of *OGDH*, *GNPNAT1*, and *CMPK1*, RSV infection was associated with significant changes in select metabolic genes only in males (Figure 4B–F). In summary, we found that genomic analysis supports metabolic assay analysis with significant sex differences in RSV phenotypes and more changes in metabolic reprogramming in male than female NAECs.

### 3.4. Incorporation of 13C Glucose in Downstream Metabolic Pathways in RSV-Infected NAECs

**Figure 5:**
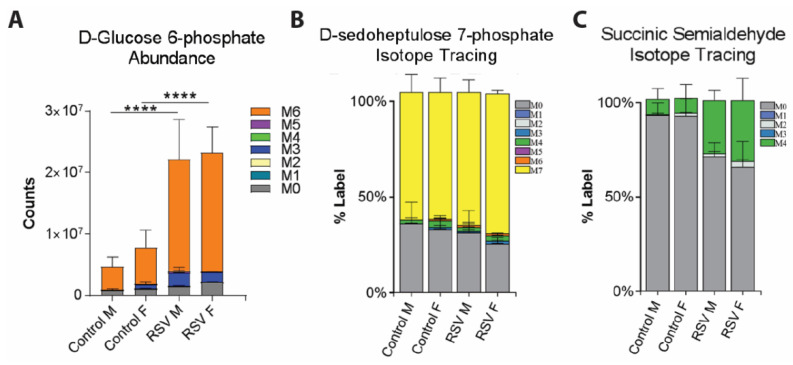
(**A**) Mass spectrometry analysis of intracellular abundance of d-glucose 6-phosphate (an immediate glucose metabolite) showing increased glycolysis in RSV group NAECs. (**B**) Isotope tracing analysis of conversion of 13C glucose to pentose phosphate pathway intermediate d-sedoheptulose 7-phosphate. Color bars indicate the proportion of metabolite derived from de novo 13C glucose uptake (indicated by number of 13C incorporated carbons (M1-M7), gray indicates metabolite production from native upstream metabolic reactions (no incorporated 13C carbons (M0)). (**C**) Higher conversion of 13C glucose to succinic semialdehyde (green bars), suggesting aberrant TCA cycle inputs in NAECs from children who were RSV infected as infants. *n* = 2 independent donors/group. **** *p* < 0.0001 by two-way ANOVA with Tukey multiple comparisons.

To gain further mechanistic insights into differential utilization of glucose by epithelial cells from children collected at age 2–3 years that had or had not been infected with RSV during infancy, we performed 13C glucose isotope tracing analysis of the NAECs in a subset of eight donors (two male children with and two male children without history of infant RSV infection; two female children with and two female children without history of infant RSV infection). In these experiments, 12C glucose (a naturally occurring glucose isotopomer) was replaced by a 13C variant of glucose (not occurring naturally) in culture media for three hours. Following the treatment, proportional incorporation of 13C carbons in downstream metabolites was measured by mass spectrometry analysis. This provided evidence for utilization of glucose in specific downstream metabolic pathways. These experiments independently confirmed significantly increased glucose uptake in NAECs from children that had been RSV infected as infants (measured as detected abundance of an early glucose metabolite, d-glucose 6-phospate, Figure 5A) but also revealed key differences in downstream utilization of glucose. Notably, in NAECs from children infected with RSV, we found greater incorporation of glucose in d-sedoheptulose 7-phosphate, which indicated higher activity of the pentose phosphate pathway (Figure 5B), and succinic semialdehyde, which suggests aberrant fueling of the TCA cycle (Figure 5C). In summary, this data further support significant durable differences in metabolism of epithelial cells from children with a history of RSV infection during infancy. We found that glucose uptake was increased in both male and female NAECs, and glucose carbons in samples from children that had been RSV infected as infants were increasingly incorporated in non-energy producing pathways, such as the pentose phosphate pathway.

## 4. Discussion

Host genetics explain only a small proportion of the heritability of asthma [11]. Early-life exposures, such as viral infections and other environmental factors, may modify an individual’s susceptibility and are likely to be more informative in our understanding of the underlying mechanisms of asthma development as well as provide targets for disease prevention. Host metabolism is sensitive to changes in diet, environment, or microbiome and represents one of the least understood early-life factors that may contribute to aberrant development of the epithelial barrier or immune skewing associated with the development of asthma [13,28]. During viral infections, infected cells need to produce high amounts of energy to support active virus replication. Therefore, viral infection potentiates cellular metabolic pathways from which necessary energy is generated [29,30,31]. Most cell energy is produced through the central carbon metabolism pathways, which include glycolysis and the tricarboxylic acid cycle (TCA cycle or citric acid cycle), generating ATP by mitochondrial oxidative phosphorylation. These pathways generate the energy necessary for the cellular processes through oxidation of glucose, glutamine, and fatty acids. Glucose is the main carbon source in the cellular environment, and multiple viral species have evolved to increase cellular uptake of glucose during infection, including rhinoviruses, human cytomegalovirus (HCMV), Epstein–Barr virus (EBV), human immunodeficiency virus (HIV), and influenza [30,32].

Our study found significant differences in the metabolism of NAECs from children with and without confirmed RSV infection in infancy. Findings in NAECs from children who had RSV during infancy included increased uptake of glucose in male and female epithelium with a likely contribution to anabolic pathways as well as increased bioenergetics and central carbon metabolism (energy-producing glycolysis and TCA cycle activity) in male epithelial cells. Importantly, epithelial cells examined in this study were sampled at least one year (at age of 2–3 years) after documented RSV infection in infancy (at age of one year), and absence of RSV infection during infancy was confirmed in the comparator group through both biweekly surveillance during RSV season and RSV serology at one year. The fact that changes in metabolism persisted in NAECs cultured one year after RSV infection in vivo strongly indicates lasting reprogramming of the airway epithelium in early life. Further epigenetic studies are needed to confirm developmental changes in regulatory gene loci associated with aberrant cellular metabolism and RSV infection. Overall, metabolism is a potent driver of epigenetic change during development [33,34]. Epigenetic reprogramming may differentially affect basal versus differentiated epithelial cell subsets, with reprogrammed basal cells further driving aberrant differentiation processes in epithelium. Therefore, increased cellular bioenergetics driven by early-life viral infection during the critical developmental period of infancy may have a lasting impact on the airway epithelium when epithelial cells are most sensitive to developmental change. As such, our findings have significant implications for developmental studies aimed at identifying mechanisms disruptive for the formation of a functionally competent airway epithelial barrier in early life.

Despite the demonstrated connection between RSV infection in infancy and epithelial reprogramming in vivo, mechanistic causality in this relationship is not clear. RSV infection is certainly sufficient to induce changes in metabolism. This is supported by studies showing increased glycolysis, pentose phosphate pathway activity, and mitochondrial respiration in RSV-infected airway epithelial cells in vitro [19,20,21] and in vivo [22]. These metabolic phenotypes largely agree with our findings of increased central energy and anabolic metabolism in primary airway cells from children with a history of RSV infection in infancy. However, the aforementioned and referenced studies were performed during active RSV infection. Whether RSV infection can promote lasting epigenetic change in airway epithelial cells remains to be demonstrated in our longitudinal studies with serial airway epithelial sampling. On the other hand, an intriguing alternative hypothesis is that altered host metabolism may represent an overlooked upstream early-life factor in driving common susceptibility to repeated or more severe RSV infection and/or wheeze and asthma development due to common disruption of normal epithelial barrier function. Such a dilemma can only be resolved by careful longitudinal cohort studies coupled with mechanistic studies of human samples aimed to resolve causality.

We found significant changes in energy-producing metabolic pathways, specifically in males. Despite demonstrated higher glucose uptake in female epithelial cells by 13C fluxomics, this was not coupled with NADH energy production. Given lack of correlation between glucose uptake and markers of glycolysis in females, it is likely that females utilize glucose in biosynthetic rather than bioenergetics reactions to a greater degree than males, which remains to be shown further in functional follow-up studies. This reinforces the study of sex as a critical biological variable in our data and the need to understand sex differences in mechanisms of RSV infection, airway epithelial development, and asthma [35,36]. Intriguingly, respiratory tract infections afflict males to a greater degree [37], including incidence and severity of RSV in the first two years of life [38]. Moreover, the prevalence of wheeze and asthma is higher in boys than girls before puberty, which reverses in adulthood—a phenomenon known as “puberty switch” [39]. Whether such sex differences in susceptibility to disease are driven or modulated by sex hormones and metabolism is an intriguing and currently unanswered question. It remains to be investigated whether RSV-linked metabolic reprogramming in infancy is causally linked to subsequent development of asthma. This is the subject of upcoming studies in longitudinal birth cohorts by our groups.

While this study has several strengths, including a study designed to specifically address the role of infant RSV infection in epithelial reprogramming and the careful surveillance and phenotyping of infant infection, we acknowledge several important limitations of our exploratory study: (1) limited sample sizes due to the technical difficulty of sampling and consistent primary expansion and culture of infant airway epithelium; (2) our metabolism assay results were not adjusted for multiple statistical testing due to small sample sizes and large variation inherent in functional studies of cellular metabolism in heterogeneous human samples. A larger mechanistic study in a different set of samples is warranted to fully profile the metabolome of RSV-infected NAECs with statistical adjustment for multiple testing and adjustment for clinical confounding variables. Nevertheless, our study represents an important step towards the characterization of NAEC metabolism associated with natural RSV infection; and (3) metabolism studies were limited to the assessment of central carbon metabolism: glycolysis and TCA intermediate metabolism. It is quite possible that there are significant differences between RSV and healthy NAECs in utilization of lipids, amino acids, or fatty acids; this remains to be tested. More careful phenotyping is needed to fully map out specific metabolic pathways.

Overall, our new data expand upon a series of significant findings that have elucidated the impact of infant RSV infection on epithelial development. In summary, our study supports the proposed phenomenon of metabolic reprogramming of epithelial cells associated with RSV infection in infancy and opens exciting new venues for pursuing mechanisms of RSV-induced epithelial dysfunction and upstream pathways that causally contribute to the development of airway disease in early life.

## Figures and Tables

**Table 1:** Comparison of demographic, biospecimen collection, and clinical characteristics of children included in this nested cohort study from among those in the larger INSPIRE cohort.

Characteristic	Nested Cohort, Children Included *N* = 24	Entire Cohort, Children Not Included *N* = 1922
Sex, % male	50% by design	52%
Birth weight (g), mean ± s.d.	3439 ± 406	3431 ± 462
Gestational age, mean ± s.d.	39.35 ± 0.89	39.13 ± 1.10
Age of first RSV infection, months, mean ± s.d.	3.9 ± 2.2	4.0 ± 2.0
Severity of first RSV infection, RSS * (mean ± s.d.)	3.1 ± 1.2	3.1 ± 2.0
Age of NAEC collection in nested cohort study (mean ± s.d.)	37.0 ± 2.0	Not applicable
1-year RSV ELISA lysate ** (median, IQR)	175 (75, 10,282)	246 (32, 2886)

* RSS, Respiratory severity score. An ordinal score from 0–12, with higher numbers indicating greater clinical severity. ** RSV ELISA lysate was measured at the one-year visit, not at a fixed time following infection. Because of this, the time between RSV infection and RSV serology varies significantly. Overall, 54% of the INSPIRE cohort was RSV serologically positive at one year.

## Data Availability

Data will be made available upon request.
